# Response Suppression Delays the Planning of Subsequent Stimulus-Driven Saccades

**DOI:** 10.1371/journal.pone.0086408

**Published:** 2014-01-22

**Authors:** Jeffrey Weiler, Trina Mitchell, Matthew Heath

**Affiliations:** 1 School of Kinesiology, The University of Western Ontario, London, Ontario, Canada; 2 Graduate Program in Neuroscience, The University of Western Ontario, London, Ontario, Canada; University of California, Davis, United States of America

## Abstract

The completion of an antisaccade selectively increases the reaction time (RT) of a subsequent prosaccade: a result that has been interpreted to reflect the residual inhibition of stimulus-driven saccade networks [1], [2]. In the present investigation we sought to determine whether the increase in prosaccade RT is contingent on the constituent antisaccade planning processes of response suppression *and* vector inversion or is limited to response suppression. To that end, in one block participants alternated between pro- and antisaccades after every second trial (task-switching block), and in another block participants completed a series of prosaccades that were randomly (and infrequently) interspersed with no-go catch-trials (go/no-go block). Notably, such a design provides a framework for disentangling whether response suppression and/or vector inversion delays the planning of subsequent prosaccades. As expected, results for the task-switching block showed that antisaccades selectively increased the RTs of subsequent prosaccades. In turn, results for the go/no-go block showed that prosaccade RTs were increased when preceded by a no-go catch-trial. Moreover, the magnitude of the RT ‘cost’ was equivalent across the task-switching and go/no-go blocks. That prosaccades preceded by an antisaccade *or* a no-go catch-trial produced equivalent RT costs indicates that the conjoint processes of response suppression and vector inversion do not drive the inhibition of saccade planning mechanisms. Rather, the present findings indicate that a general consequence of response suppression is a residual inhibition of stimulus-driven saccade networks.

## Introduction

The most frequent motor actions that humans make are saccades with direct stimulus-response compatibility (i.e., prosaccades). Importantly, the dimensional overlap between stimulus and response allows for the mediation of a prosaccade via stimulus-driven and retinotopically organized motor maps in the superior colliculus [3]. In contrast, antisaccades entail the intentional process of decoupling the spatial relations between stimulus and response and require the evocation of a saccade to a target’s mirror-symmetrical location (i.e., 180° spatial transformation). As such, contrasting pro- and antisaccades provides a basis for understanding how top-down and cognitive control influences the oculomotor system. Indeed, an extensive literature has shown that antisaccades produce longer reaction times (RT) [4], [5], increased directional errors [4], and less accurate and more variable endpoints [6] than their prosaccade counterparts. These behavioural ‘costs’ have been attributed to a two-component process requiring: (1) the suppression of a stimulus-driven prosaccade (i.e., response suppression), and (2) the visual remapping of a target’s spatial properties to mirror-symmetrical space (i.e., vector inversion) [7]. Moreover, neuroimaging and electrophysiological evidence from humans and non-human primates has shown that the preparatory period of antisaccades is associated with increased activity in the ‘classic cortical saccade network’ (i.e., frontal eye field, supplementary eye field, and lateral intraparietal area) [8], [9], [10], [11], as well as a respective increase and decrease in collicular fixation and build-up neurons [12], [13]. According to Brown et al. [8], the modulation of oculomotor networks during the antisaccade task represents an oculomotor pre-setting that is designed to inhibit the evocation of a stimulus-driven prosaccade at target onset (i.e., the visual grasp reflex: [14]). In other words, pre-setting serves as cortical-based inhibition of the baseline firing rates of saccade neurons.

Recently, our group has shown that a corollary of antisaccade pre-setting is a residual inhibition of stimulus-driven oculomotor networks [1], [2], [15]. In addressing this issue, participants alternated between pro- and antisaccades using a classic task-switching schedule (i.e., AABB) as well as a pseudo-randomized task-switching schedule (i.e., AABAABB…). Results for both schedules showed that prosaccades preceded by an antisaccade (i.e., task-switch prosaccade) elicited longer RTs than prosaccades preceded by their same task counterparts (i.e., task-repetition prosaccade). In contrast, antisaccades preceded by a prosaccade (i.e., task-switch antisaccade) yielded RTs that were comparable to antisaccades preceded by their same task counterparts (i.e., task-repetition antisaccades). In other words, the completion of an antisaccade imparts a residual inhibition that delays the planning of a to-be-completed prosaccade: a result our group has referred to as the unidirectional prosaccade switch-cost. As well, the prosaccade switch-cost has been shown to selectively manifest following a correct antisaccade (i.e., a response planned mirror-symmetrical to the target) but not an error antisaccade (i.e., a saccade initially, and incorrectly, directed at the veridical target location) [16]. Indeed, that correct - but not error - antisaccades were tied to a prosaccade switch-cost suggests that the constituent elements associated with the *planning* of a correct antisaccade engenders a residual level of oculomotor inhibition that delays the *planning* of a subsequent prosaccade.

An important issue to address is whether the unidirectional prosaccade switch-cost is contingent upon the constituent planning processes of response suppression *and* vector inversion or is limited to response suppression. The basis for this question stems from a countermanding study by Pouget et al. [17] showing that stimulus-driven prosaccades completed after a successful stop-signal saccade are associated with a delay in the onset of saccade neuron activity in the frontal eye-fields and superior colliculus. In other words, Pouget et al. found that inhibiting a prosaccade leads to a residual inhibition of oculomotor planning networks. Thus, the present investigation sought to determine whether the unidirectional prosaccade switch-cost is a specific consequence of the antisaccade task (i.e., response suppression and vector inversion) or represents a more general phenomenon associated with response suppression. In accomplishing our objective, we had participants alternate between pro- and antisaccades using the task-switching schedule (i.e., AABB; task-switching block) employed in our group’s previous work [1], [2], [15], and in a separate block required that participants complete a series of prosaccades that were randomly interleaved with no-go catch-trials (i.e., go/no-go block). Most importantly, we were interested in contrasting the putative changes in prosaccade RT when preceded by an antisaccade and a no-go catch-trial. Indeed, if the conjoint process of response suppression and vector inversion engenders a residual level of oculomotor inhibition then a selective lengthening of prosaccade RTs should be observed when preceded by an antisaccade but not when preceded by a no-go catch-trial. In contrast, if response suppression alone is responsible for a residual level of oculomotor inhibition then a lengthening of prosaccade RTs should be observed when preceded by either an antisaccade or a no-go catch-trial. Further, if the latter prediction proves correct then a direct comparison of the magnitude of the prosaccade RT lengthening may provide a basis for determining whether common or dissociable mechanisms contribute to the residual inhibition of oculomotor planning mechanisms.

## Methods

### Participants

Seventeen participants (7 male, 10 female; age range = 18–20 years) from the University of Western Ontario community volunteered for the current investigation. All participants declared being right-hand dominant and had normal or corrected-to-normal vision. Prior to data collection participants provided informed written consent. This study was approved by the Office of Research Ethics, the University of Western Ontario, and was conducted in accordance with the Declaration of Helsinki.

### Apparatus and Procedures

Participants sat at a table with their head stabilized via a head-chin rest for the duration of data collection. Visual stimuli were presented on a 30-inch LCD monitor (60 Hz, 8 ms response rate, 1280 by 960 pixels, Dell 3007WFP, Round Rock, TX, USA) centered on the participant’s midline and located at a viewing distance of 550 mm. The gaze location of the participant’s left eye was obtained via a video-based chin-mounted eye tracking system (Eye-Trac 6: Applied Sciences Laboratories, Bedford, MA, USA) sampling at 360 Hz. Prior to data collection a nine-point calibration of the participant’s viewing space was performed. Two additional monitors that were only visible to the experimenter provided: (1) real-time point of gaze information, (2) a visual depiction of trial-to-trial saccade trajectories (e.g., displacement, velocity), and (3) information about the accuracy of the eye tracking system (i.e., to determine a necessary recalibration or drift correction). All computer events and visual stimuli were controlled via MATLAB (7.6: The Math Works, Natick, MA, USA) and the Psychophysics Toolbox extensions version 3 [18]. The lights in the experimental suite were extinguished during data collection.

Visual stimuli were presented against a high contrast black background. Stimuli included a green and a red fixation cross (1.0°) that were centered horizontally on the monitor and at the eye-level of the participant. In addition, yellow crosses (1.0°) served as targets and were located 10.5° (proximal) or 15.5° (distal) left and right of the fixation cross. All trials commenced with the presentation of the green or the red fixation cross which alerted participants to direct their gaze to its location. After a stable gaze was achieved (±1.5° for 500 ms), a randomized foreperiod (1,000–2,000 ms) was introduced during which time the fixation cross remained visible (i.e., no-gap paradigm). Following the foreperiod, the fixation cross was removed and a target stimulus was briefly presented (i.e., 50 ms) in one of the four target locations (i.e., combination of visual space by target eccentricity).

**Figure 1:**
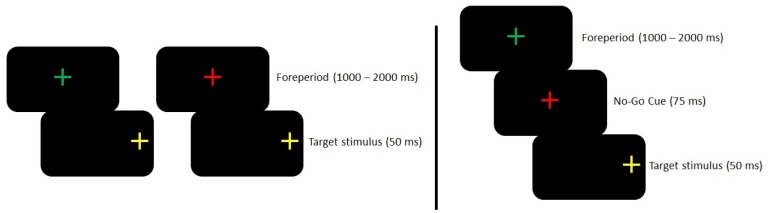
Schematic of visual event for pro- and antisaccades in the task-switching block (left of solid line) and no-go trials in the go/no-go block (right of solid line). In the task-switching block green or red fixation cross was visible for a variable foreperiod after which time one of four possible target stimuli was briefly presented. The green and red fixation cross denoted a saccade to the target’s veridical (i.e., prosaccade) or mirror-symmetrical (i.e., antisaccade) location, respectively. In the go/no-go block, prosaccade trials were the same as they were in task-switching block. For no-go trials, following the foreperiod the green fixation cross was replaced with a red fixation cross for 75 ms prior to target onset which signaled participants to withhold a prosaccade.

Participants completed two blocks of trials. In one block (i.e., task-switching block), participants alternated between pro- and antisaccades after every second trial (i.e., AABB). Notably, the green fixation cross informed participants to saccade to the veridical target location (i.e., prosaccade), whereas the red fixation cross indicated a saccade to the target’s mirror-symmetrical location (i.e., antisaccade) (see Figure 1). The trial-to-trial target locations (i.e., proximal or distal eccentricity in left or right visual field) were randomly selected. Participants’ responses were categorized as a task-switch (i.e., prosaccade preceded by an antisaccade, or vice versa) or a task-repetition (i.e., pro- or antisaccade preceded by the same task) pro- and antisaccade responses. Each of the four aforementioned trial-types (i.e., task-switch and task-repetition prosaccades; task-switch and task-repetition antisaccades) comprised 36 trials (or 25%) of the 144 trials in the task-switching block. The presentation of the target (and fixation cross removal) cued participants to make their response as “quickly and accurately as possible”. The task (i.e., pro-, antisaccade) associated with the first trial in this block was counterbalanced across participants. Trials where a directional error was committed (i.e., prosaccade instead of the instructed antisaccade, or vice versa) were not analyzed. In addition, as the first trial in this block was neither a task-switch nor a task-repetition trial, it was excluded from subsequent analysis.

In the other block (i.e., go/no-go block), participants completed a series of prosaccades randomly interspersed with a series of no-go catch-trials. This block included the same visual stimuli presentation as prosaccades in the task-switching block; that is, a green fixation cross was presented for a variable foreperiod after which time a target was presented (and fixation cross was extinguished) in one of the four potential locations. Importantly, 28 of the 196 trials in this block (14.3%) entailed a situation wherein the green fixation cross was replaced with the red fixation cross 75 ms prior to target onset, and the fixation colour-change instructed participants to withhold their response (i.e., no-go catch-trial; see Figure 1). Notably, 14.3% of total trials were used as no-go catch-trials as this frequency is similar to other investigations which have used no-go trials to examine top-down inhibitory control [19], [20]. In addition, our pilot testing demonstrated that a no-go cue occurring either 25 or 50 ms prior to target onset resulted in participants failing to withhold a saccade on 55% and 42% of all no-go trials, respectively. Thus, in order for participants to successfully complete the no-go task but still supress an expectant saccade (i.e., context-dependent response suppression; [21]), we employed a no-go cue 75 ms prior to target onset. In terms of trial-types, responses were categorized as standard prosaccades (i.e., a prosaccade preceded by a prosaccade) or post catch-trial prosaccades (i.e., prosaccade preceded by a no-go catch-trial). Trials where an error was committed (i.e., a failure to withhold a response during a no-go trial, or no response on a prosaccade trial) were not analyzed and were inserted back into the trial matrix. Recall that in this block we sought to determine the effect of response suppression on a subsequent prosaccade trial. As such, prosaccade completed after an error no-go catch-trial (i.e., failure to suppress a response) were not included in subsequent analyses.

The ordering of the task-switching and the go/no-go blocks was counterbalanced across participants and the different blocks were completed on separate days separated by 24 hours. The separate sessions were used to prevent participants from experiencing mental and/or eye fatigue.

### Data Reduction

Displacement data were filtered offline using a dual-pass Butterworth filter employing a low-pass cut-off frequency of 15 Hz. Filtered displacement data were used to compute instantaneous velocities via a five-point central finite difference algorithm. Acceleration data were computed similarly via the velocity data. Saccade onset was determined on the basis of velocity and acceleration values that exceeded 30°/s and 8,000°/s^2^, respectively. Reaction time (RT) was computed as the time between target presentation and saccade onset. Means and within-participant 95% confidence intervals [22] are reported below.

## Results

### Alternating between Pro- and Antisaccades: The Unidirectional Prosaccade Switch-cost

**Figure 2:**
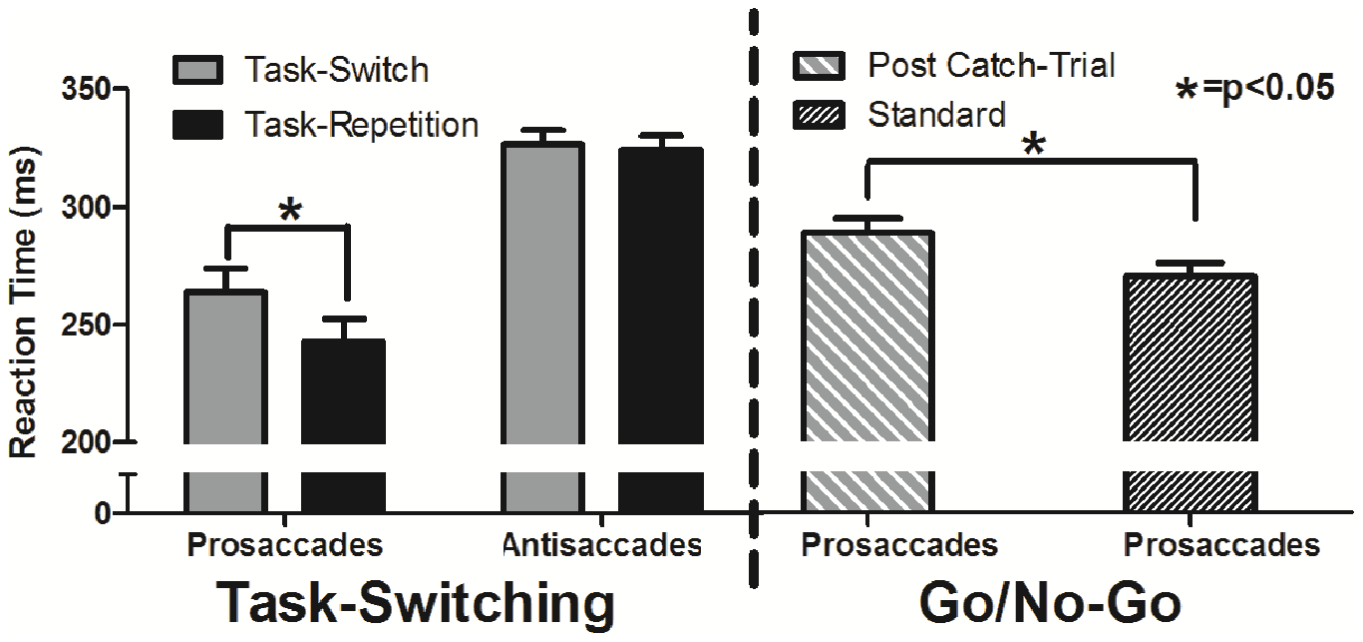
Mean reaction times for the task-switching (left) and go/no-go (right) block. Error bars represent 95% within-participant confidence intervals.

To determine if alternating between pro- and antisaccades resulted in the unidirectional prosaccade switch-cost, RTs associated with the task-switching block were examined via 2 (task: pro-, antisaccade), by 2 (task transition: task-switch, task-repetition) fully repeated measures ANOVA. Results yielded main effects of task, F(1,16) = 95.51, p<0.001, task transition, F(1,16) = 9.29, p<0.01, and their interaction, F(1,16) = 5.78, p<0.05. Task-switch prosaccades produced longer RTs (264 ms, CI_95%_ = 10) than their task-repetition counterparts (243 ms, CI_95%_ = 10), t(16) = 3.26, p<0.01, whereas task-switch (326 ms, CI_95%_ = 6) and task-repetition (324 ms, CI_95%_ = 6) antisaccades did not reliably differ, t(16) = 0.54, p = n.s. (Figure 2). Thus, results demonstrate the unidirectional prosaccade switch-cost. Additionally, we submitted the number of saccade directional errors to the same ANOVA model identified above and observed a main effect of task, F(1,16) = 35.51, p<0.001: prosaccades elicited fewer directional errors (1.8, CI_95%_ = 0.95) than antisaccades (5.5, CI_95%_ = 0.95).

### No-go Catch-trials Delay the Planning of Subsequent Prosaccades

In the go/no-go block participants were periodically required to inhibit a prosaccade in response to an infrequent no-go stimulus. To determine whether a no-go catch-trial influenced the planning time for a subsequent prosaccade we contrasted the RTs of standard and post catch-trial prosaccades. Results demonstrated that standard prosaccades (268 ms, CI_95%_ = 6) had shorter RTs compared to their post catch-trial counterparts (286, CI_95%_ = 6), t(16) = 4.58, p<0.001, (Figure 2). Further, and in line with the task-switching block, we sought to provide a measure of error trials. Recall, however, that error trials in this block (i.e., a failure to withhold a response during a no-go trial, or no response on a standard prosaccade trial) were placed back into the randomized trial matrix. Indeed, we adopted such a strategy to ensure that a sufficient number of post catch-trial prosaccades were preceded by a successful no-go catch-trial. Thus, the total number of attempted no-go catch-trials differed across participants (range = 29–54 trials). Given the between-participant differences in number of attempted no-go catch-trials we elected to provide a qualitative analysis of error rates in the go/no-block. Results indicated that catch-trials (9.8) were associated with more errors than standard (0.8), or post-catch trial (1.4) prosaccades.

### Equivalent Between-block Effects of Suppressing a Stimulus-driven Prosaccade

Results thus far have demonstrated that prosaccades preceded by either an antisaccade or a no-go catch-trial elicited a RT cost. Specifically, task-switch prosaccades showed a 21 ms increase in RT compared to their task-repetition counterparts, whereas post catch-trial prosaccades yielded an 18 ms increase in RT compared to their standard prosaccade counterparts. Here we compared participant-specific RT difference scores for the task-switching (i.e., task-switch prosaccade minus task-repetition prosaccade) and go/no-go (i.e., post-catch trial prosaccade minus standard prosaccade) blocks to determine whether the magnitude of the RT costs differed between blocks. The results of this analysis yielded a null between-block cost, t(16) = 0.51, p = n.s.

## Discussion

A general finding from the task-switching block was that antisaccades produced longer RTs and elicited more directional errors than prosaccades. Such results are in accord with extensive work demonstrating that the constituent components of the antisaccade task (i.e., response suppression and vector inversion) represent time-consuming and measureable processes [7]. In addition, RTs for prosaccades, but not antisaccades, were found to be influenced by the nature of the preceding task. In particular, task-switch prosaccades produced longer RTs than their task-repetition counterparts, whereas RTs for task-switch and task-repetition antisaccades did not reliably differ. In other words, results provide a faithful replication of the unidirectional prosaccade switch-cost [1], [2], [15], [16], [23]. As indicated in the Introduction, such a switch-cost has been interpreted to reflect that the constituent components of the antisaccade task engender a residual level of inhibition that reduces the efficiency of the oculomotor networks supporting the planning of stimulus-driven prosaccades.

In the go/no-go block participants completed a series of prosaccade trials that involved the infrequent and random occurrence of no-go catch-trials. A general finding from this block was that participants elicited a larger number of errors for no-go catch-trials compared to post catch-trial prosaccades or standard prosaccades. However, and what is more germane to our investigation, is the observation that RTs for post catch-trial prosaccades were longer than their standard prosaccade counterparts. As well, the RT cost associated with the go/no-go block (i.e., post catch-trial prosaccade minus standard prosaccade) was commensurate to that observed in the task-switching block (i.e., task-switch prosaccade minus task-repetition prosaccade). Further, *a posteriori* correlation revealed that the magnitude of the switch-cost between the task-switching and no-go blocks approached conventional levels of significance (r = 0.43, p = 0.08). Notably, such a result indicates a consistent inhibitory cost on prosaccade planning networks independent of whether the previous trial was an antisaccade or no-go catch-trial. Taken together, the present results counter the assertion that the constituent elements of the antisaccade task (i.e., response suppression and vector inversion) delay the planning of a subsequent prosaccade. Rather, the current findings indicate that a general consequence of supressing a prosaccade is a residual inhibition of stimulus-driven saccade networks.

It should be noted that the assertion that response suppression selectively inhibits prosaccade planning mechanisms is not entirely at odds with our group’s previous work. For example, DeSimone et al. [16] showed that correct antisaccades, but not error antisaccades (i.e., a saccade initially, and incorrectly, directed at the veridical target location), were associated with a delay in the planning time for a subsequent prosaccade. As mentioned in the Introduction, such a result was interpreted to reflect that the conjoint process of response suppression *and* vector inversion contribute to the residual inhibition of stimulus-driven saccade networks. However, DeSimone et al’s work was not designed to disentangle the putative consequence of response suppression and vector inversion. As a result, an alternative explanation of that work is that error antisaccades do not impart a prosaccade switch-cost because such actions are not associated with the suppression of a stimulus-driven prosaccade. Moreover, it is important to recognize that several studies have documented residual inhibition of oculomotor planning networks when a signal to withhold a saccade is provided after target onset (i.e., stop-signal paradigm). For example, Emeric et al. [24] had participants perform a series of prosaccades wherein a stop-signal was provided infrequently (and randomly) during a response’s RT interval. Results showed that inhibiting the execution of a planned prosaccade was associated with an increase in RT on the subsequent prosaccade trial. In addition, Pouget et al’s [17] study of non-human primates interleaved prosaccade trials with infrequent stop-signal trials while concurrently recording the activity of saccade neurons in the frontal eye field and superior colliculus. Their results demonstrated that the successful suppression of a stimulus-driven saccade led to a 17 ms increase in the RT of the subsequent prosaccade: a result that mirrors the respective 21 ms and 18 ms RT costs associated with the task-switching and go/no-go blocks used in the current investigation. Moreover, the RT cost in Pouget et al’s study was associated with a delay in the onset of activity of frontal eye field and collicular saccade neurons. Thus, the above-mentioned work in combination with the present results indicate that a consequence of suppressing a stimulus-driven prosaccade is a transient delay in the onset of neural activity associated with the planning of a subsequent prosaccade.

Our interpretation of the present results requires that two issues be addressed. First, trials in the task-switching and go/no-go blocks differed with respect to the time participants were afforded to suppress an upcoming stimulus-driven saccade. Recall that during the task-switching block a red fixation cross presented between 1,000 and 2,000 ms prior to target onset alerted participants of the need to suppress a stimulus-driven saccade; that is, the red fixation cross indicated an antisaccade trial. In contrast, the go/no-go block provided a cue to suppress a stimulus-driven saccade (via a green to red fixation colour-change) 75 ms prior to target onset. As such, between-block temporal differences related to advanced knowledge of response suppression may have led to between-block differences in the inhibition of prosaccade planning mechanisms. A priori, we considered equating for this between-block difference by providing the no-go cue for the same duration as the advanced cuing procedure used in the antisaccade task (i.e., 1,000–2,000 ms in advance of target onset). Importantly, however, neuroimaging work has shown that providing a no-go cue well in advance of response cuing (e.g., 2,000–7,000 ms) does not result in context-dependent response suppression that is observed in the antisaccade task [21]. Such a finding reflects that the neural activity associated with planning a movement (e.g., stimulus-driven prosaccade) dissipates when the choice to perform an alternate response (e.g., remain fixated) is selected [25]. In other words, providing a cue mitigating the need to plan an active response disrupts the normal planning mechanisms of stimulus-driven saccade networks [26]. In order to ameliorate this issue and require that participants engage in context-dependent response suppression, we elected to provide the no-go cue 75 ms to target onset. Indeed, that participants elicited an appreciable number of errors on no-go catch-trials as compared to antisaccade trials indicates that: 1) such trials involved classic prosaccade planning, and 2) the successful completion of a no-go catch-trial was contingent on the top-down process of response suppression. It is worth commenting that the commensurate RT cost between the task-switching and go/no-go blocks may be specific to the 14% no-go catch-trial frequency and no-go cuing (i.e., 75 ms prior to target onset) used in the present investigation. However, what is most notable from the current results is that our findings demonstrate that context-dependent response suppression engenders a residual inhibition to stimulus-driven saccade networks. The second issue to address relates to the fact that task-repetition prosaccades from the task-switching block and standard prosaccades in the go/no-go block represent the exemplar tasks by which responses preceded by a trial requiring response suppression (i.e., task-switch prosaccades and post catch-trial prosaccades) were compared. However, inspection of Figure 2 and *a posteriori* analyses indicated that RTs for task-repetition prosaccades (243 ms, CI_95%_ = 7) were shorter than the standard prosaccades used in the go/no-go block (268 ms, CI_95%_ = 7), t(16) = 5.55, p<0.01, and within-participant RT variability was reduced in the former (51 ms, CI_95%_ = 4) as compared to the latter (59 ms, CI_95%_ = 4) trial-type, t(16) = 2.20, p<0.05. That standard prosaccades were associated with longer and more variable RTs is attributed to the increased level of response uncertainty in the go/no-go block [27], [28]. Indeed, in the task-switching block participants were explicitly aware of the required response well in advance of target onset, whereas the appropriate response cue (i.e., go versus no-go) was provided to participants only 75 ms prior to target onset in the go/no-go block. In spite of this between-block difference, it is important to recognize that the level of response uncertainty did not influence the magnitude by which response suppression for an antisaccade or a no-go catch-trial delayed the planning times for a subsequent prosaccade. After all, the present findings demonstrate that the magnitude of the response suppression RT cost did not reliably differ across the task-switching and go/no-go blocks. Such a finding suggests that the residual inhibition engendered by response suppression is a phenomenon that needs to be overcome independent of the required planning time associated with a stimulus-driven prosaccade.

### Conclusions

Our results show that the successful completion of an antisaccade or a no-go catch-trial results in a comparable increase in the RT of a subsequent prosaccade. Thus, we conclude that the antisaccade task does not uniquely impede the planning of a subsequent prosaccade; rather, we propose that response suppression imparts a residual inhibition of the oculomotor networks supporting stimulus-driven prosaccades.
